# Atomic-scale modelling of organic matter in soil: adsorption of organic molecules and biopolymers on the hydroxylated α-Al_2_O_3_ (0001) surface

**DOI:** 10.1098/rsta.2022.0254

**Published:** 2023-07-10

**Authors:** Aneesa Ahmad, Natalia Martsinovich

**Affiliations:** Department of Chemistry, University of Sheffield, Sheffield, UK

**Keywords:** soil organic carbon, minerals, alumina, hydroxylated surface, adsorption, polysaccharides

## Abstract

Binding of organic molecules on oxide mineral surfaces is a key process which impacts the fertility and stability of soils. Aluminium oxide and hydroxide minerals are known to strongly bind organic matter. To understand the nature and strength of sorption of organic carbon in soil, we investigated the binding of small organic molecules and larger polysaccharide biomolecules on α-Al_2_O_3_ (corundum). We modelled the hydroxylated α-Al_2_O_3_ (0001) surface, since these minerals' surfaces are hydroxylated in the natural soil environment. Adsorption was modelled using density functional theory (DFT) with empirical dispersion correction. Small organic molecules (alcohol, amine, amide, ester and carboxylic acid) were found to adsorb on the hydroxylated surface by forming multiple hydrogen bonds with the surface, with carboxylic acid as the most favourable adsorbate. A possible route from hydrogen-bonded to covalently bonded adsorbates was demonstrated, through co-adsorption of the acid adsorbate and a hydroxyl group to a surface aluminium atom. Then we modelled the adsorption of biopolymers, fragments of polysaccharides which naturally occur in soil: cellulose, chitin, chitosan and pectin. These biopolymers were able to adopt a large variety of hydrogen-bonded adsorption configurations. Cellulose, pectin and chitosan could adsorb particularly strongly, and therefore are likely to be stable in soil.

This article is part of a discussion meeting issue ‘Supercomputing simulations of advanced materials’.

## 1. Introduction

The α-Al_2_O_3_ mineral (corundum) has been extensively studied because of its use in a wide range of technological applications, such as thin film substrates and catalyst supports [1,2]. Moreover, α-Al_2_O_3_, as well as aluminium hydroxide and the related aluminosilicate minerals, commonly occurs in rocks and soil [3]. In particular, the presence of aluminium oxides and hydroxides in soil has been linked to the stability of soil organic carbon [4]. Presence of organic carbon, such as humin, lignin and polysaccharides, in soil is essential for the stability and fertility of soils; moreover, by storing carbon, soils prevent its release into the atmosphere as CO_2_ [5–7]. The main process responsible for degradation and loss of carbon from soil is microbial respiration, which results in the estimated 6 × 10^13 ^kg of carbon per year released in the atmosphere as CO_2_ [5]. Sorption of organic matter on surfaces of minerals, such as aluminium oxides and hydroxides, was found to stabilize it against microbial decay [4,8,9]. Therefore, to understand the stability of organic carbon in soil, it is essential to investigate organic molecules’ interaction with aluminium oxide surfaces.

The (0001) surface of α-Al_2_O_3_ is the most energetically stable surface of this mineral. A number of studies investigated the properties of this surface, such as the surface structure [10–15], energy [10,16] and adsorption of water [12–28] and other small molecules [29–32]. The single aluminium terminated surface is accepted as the most stable form of the bare α-Al_2_O_3_ (0001) surface [10,11,14,16]. The bare surface readily reacts with water, which dissociates to form surface hydroxyls [17,19,20,28]. The dissociation of water molecules produces two different types of surface hydroxyl groups: the OH_ads_ surface hydroxyl where the oxygen atom comes from water and the O_s_H hydroxyl where the oxygen atom is from the surface [19]. Existence of hydroxyl-terminated α-Al_2_O_3_ (0001) surfaces has been confirmed by a variety of experimental observations using thermal desorption [12], X-ray photoelectron spectroscopy (XPS) [13], atomic force microscopy [15] and vibrational spectroscopy [20]. Different types of hydroxyl terminations have been observed, depending on the humidity of the environment and the sample preparation, from the partially hydroxylated Al-terminated surface to the fully hydroxylated ‘gibbsite-like’ Al(OH)_3_-terminated surface [12,28]. While the soil environment is much less well-controlled than the ultra-high vacuum or low vapour pressure environment where spectroscopic or microscopic characterization of surfaces is carried out, it is clear that the α-Al_2_O_3_ (0001) surface is likely to be hydroxylated in the ambient moisture-containing environment, but the exact nature of hydroxylation is harder to determine experimentally, and requires complementary theoretical investigations.

Density-functional theory (DFT) simulations described hydroxylation of the α-Al_2_O_3_ (0001) surface as a multi-step process, with the first step being water dissociation at undercoordinated surface Al sites with a relatively low activation barrier, to form an OH adsorbed at the Al site and an H adsorbed at a nearby O site [19,25]. This process can be followed by subsequent water molecules dissociating at hydroxylated Al sites with higher activation barriers, eventually releasing the hydroxylated Al atoms from the surface to form the fully hydroxylated ‘gibbsite-like’ Al(OH)_3_-terminated surface, which can be described as hydrogenated O-terminated surface [17,19]. First-principles molecular dynamics simulations of alumina/water interfaces have also demonstrated the dynamic behaviour of the hydroxyl groups at the interface with liquid water [22,23], and revealed the presence of strongly bound ‘ice-like’ water molecules and weakly bound ‘liquid-like’ water molecules at the alumina (0001)/water interface [21,24], consistent with vibrational spectroscopy. *Ab initio* thermodynamics modelling showed that the gibbsite-like fully hydroxylated surface is the most stable form at high water vapour pressures, while at low water vapour pressures the surface with water molecules dissociated at Al sites is stable [16,19]. Since the first step in the hydroxylation process has a small activation barrier and can occur rapidly at ambient temperatures [17], the α-Al_2_O_3_ (0001) surface is likely to exist in the form produced in the first hydroxylation step, i.e. water dissociated at Al sites [22], in ambient environments, such as naturally occurring soils with moderate moisture content.

Although the initial water dissociation step and composition of the α-Al_2_O_3_ (0001) surface in the presence of water have received a great deal of attention, very few studies have been carried out on the adsorption of molecules on the hydroxylated α-Al_2_O_3_ (0001) surface. In particular, Yeh *et al*. [29] modelled the adsorption of catechol and other phenols on the bare and hydroxylated α-Al_2_O_3_ (0001) surface and at the alumina/water interface using force-field molecular dynamics simulations; while Poberžnik & Kokalj [30] modelled the adsorption of a silanol molecule on several hydroxylated alumina surfaces. Blanck *et al*. [31] investigated the adsorption of a series of small organic molecules on the dry and hydroxylated γ-Al_2_O_3_ (100) and α-Fe_2_O_3_ (0001) surfaces, and found correlation between the energies of adsorption on the dry and hydroxylated alumina and hydroxylated haematite surfaces. To understand the binding of organic matter in soil, further systematic studies of adsorption of organic molecules on strongly binding minerals, such as alumina, are needed.

In this work we used DFT calculations to investigate the adsorption of several organic molecules and biopolymers that are likely to be present in soil, on the hydroxylated α-Al_2_O_3_ (0001) surface as the model aluminium oxide mineral surface under environmental conditions, to determine the strength and nature of adsorption of these molecules on this hydroxylated surface. This follows on from our earlier study of adsorption of organic molecules on the bare α-Al_2_O_3_ (0001) surface [32]. The hydroxylated α-Al_2_O_3_ (0001) surface is a more realistic model of the alumina surface in soils than the bare surface, because of the inherent presence of moisture in soil. Here, we modelled the adsorption of small organic molecules that contain functional groups (alcohol, amine, amide, ester and carboxylic acid) that are typically present in soil organic matter. We then modelled the adsorption of polysaccharide biopolymers, such as cellulose, pectin, chitin and chitosan, as more realistic representations of soil organic matter adsorbed on the alumina mineral surface.

## 2. Computational method

The calculations were performed using DFT within the CP2K software package (v.7.1) [33–35], using the generalized gradient approximation (GGA) PBE exchange-correlation functional [36] with Grimme's D3 dispersion correction [37]. CP2K uses a dual basis of localized atom-centred Gaussian orbitals and plane waves. Our calculations used localized double-ζ basis sets with diffuse and polarization functions (DZVP) optimized for use in CP2K [38], and Goedecker–Teter–Hutter (GTH) pseudopotentials [39]; the plane wave cut-off energy was 400 Ry. All calculations were done at the Γ k-point. Optimization was carried out using the Broyden–Fletcher–Goldfarb–Shanno (BFGS) algorithm, with the convergence criteria of 3 × 10^−3 ^Bohr for the maximum geometry change, 4.5 × 10^−4 ^Ha Bohr^−1^ for the maximum force, 1.5 × 10^−3^ Bohr for the root mean square geometry change and 3 × 10^−4 ^Ha Bohr^−1^ for the root mean square force. The orbital transformation (OT) method was used for SCF diagonalization, with a DIIS minimizer, preconditioner FULL_SINGLE_INVERSE and an estimate for the energy gap of 0.1 Ha in preconditioning. Grimme's D3 method with zero damping was used, with *s*_6_, *s_r_*_,6_ and *s*_8_ coefficients from the University of Bonn web page [40], as implemented in CP2K.

**Figure 1.**
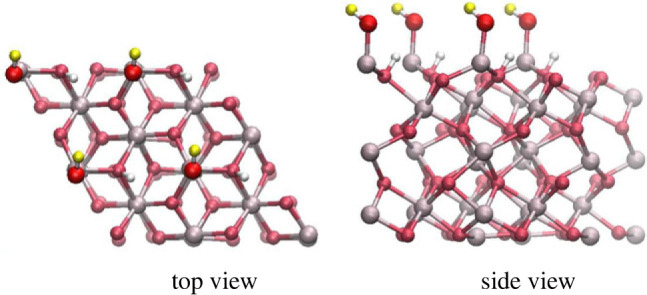
Top view and side view of the α-Al_2_O_3_ (0001)-oriented 2 × 2-extended hydroxylated slab. Light grey spheres—Al atoms, dark red—O atoms of α-Al_2_O_3_, bright red—O atoms of the top surface hydroxyl layer, yellow—H atoms of the top surface hydroxyl layer, white—H atoms attached to O subsurface atoms of α-Al_2_O_3_ and forming a lower layer of hydroxyls. (Online version in colour.)

The α-Al_2_O_3_ (0001)-oriented hydroxyl-terminated surface was modelled using periodic slabs, with 12 Al and O atomic layers in the slab, and a layer of hydroxyls terminating the top of the slab (figure 1). This slab thickness was found to be converged in our previous study of single Al-terminated (0001)-oriented α-Al_2_O_3_ slabs [32]. To create the hydroxylated surface, the undercoordinated Al atoms on the surface of the single Al-terminated (0001) α-Al_2_O_3_ slabs were terminated with hydroxyl groups, while the undercoordinated O atoms in the layer just below the surface Al atoms were terminated by hydrogen atoms (figure 1), forming the structure produced in the first step of hydroxylation by water molecules dissociating at Al sites [16–18]. In this structure, two hydroxyls were formed per each Al atom, equivalent to dissociating one water molecule per Al atom and adsorbing both the water hydroxyl and the water proton on the surface. Thus, there were two types of surface hydroxyls present on the surface: those terminating the Al atoms and forming the top layer of hydroxyls (with O shown as bright red spheres and H shown as yellow spheres in figure 1), and those terminating subsurface O atoms (with H atoms shows as white spheres in figure 1).

Adsorption of small molecules was modelled using slabs 2 × 2 extended in the horizontal dimensions to accommodate the adsorbate. These slabs had a hexagonal surface unit cell with the lattice parameter of 9.575 Å and contained 92 atoms (32 Al, 52 O and eight H atoms). Larger slabs, 6 × 6 extended in the horizontal dimensions and consisting of 828 atoms (288 Al, 468 O and 72 H atoms; hexagonal surface unit cell with the lattice parameter of 28.726 Å) were used to model the adsorption of biopolymers. The slab lattice parameters were fixed at their bulk values obtained in our earlier study [32]. The cell height was 50 Å, so that the atoms in image cells were separated in the vertical direction by 35–40 Å of vacuum (depending on the size of the adsorbate). Adsorbates were placed on one side of the slab, and all atoms were fully optimized.

Adsorption energies (*E*_ads_) were calculated using the following equation:

2.1 $$E_{\text{ads }}=E_{\text{slab + molecule}}-(E_{\text{slab}}+E_{\text{molecule}}),$$

where $E_{\text{slab + molecule}}$, $E_{\text{slab}}$ and $E_{\text{molecule}}$ are the total energies of the slab with adsorbate, the slab and the isolated molecule, respectively. The calculated adsorption energies of small molecules were corrected for the basis set superposition error (BSSE) using the counterpoise method [41]. The BSSE corrections for adsorbed small molecules were very small, between 0.01 and 0.08 eV. The BSSE corrections for adsorbed biopolymers (calculated for several selected configurations of each adsorbate) were also very small, between 0.05 and 0.17 eV (5–10% of the total adsorption energies); therefore, the adsorption energies of the biopolymers were reported without correction.

To investigate the possible effects of the density functional and of the method for describing van der Waals interactions, the lowest-energy adsorption configurations for small molecule adsorbates were additionally calculated using three alternative methods: PW91 functional [42] with the same D3 dispersion correction (optimization and adsorption energies calculations using CP2K v.8.2), the non-local vdW-DF2 functional [43] (optimization and adsorption energies calculations) and B3LYP functional [44] with the D3 dispersion correction (adsorption energies calculations using vdW-DF2 geometries without further optimization).

## 3. Results and discussion

### (a) Adsorption of small organic molecules on the hydroxylated α-Al_2_O_3_ (0001) surface

**Figure 2.**
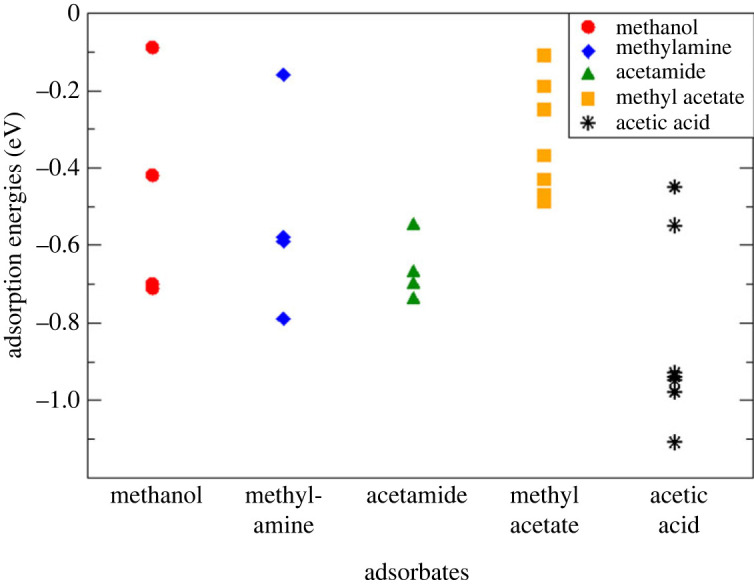
Adsorption energies of all adsorbed configurations of methanol, methylamine, acetamide, methyl acetate and acetic acid molecules on the hydroxylated α-Al_2_O_3_ (0001) surface. (Online version in colour.)

As the first stage in investigating the binding of organic carbon on the hydroxylated alumina mineral in soil, we modelled the adsorption of several small organic molecules on the hydroxylated α-Al_2_O_3_ (0001) surface, to investigate the nature and strength of different functional groups' binding to this surface. We selected organic molecules containing functional groups that can be present in polysaccharides in soil: alcohol, amine, amide, ester and carboxylic acid. Since there are many possible adsorption configurations on this hydroxylated surface because of multiple possibilities of forming surface–adsorbate hydrogen bonds, an exhaustive search for all possible adsorption configurations is not feasible. Therefore, we investigated a set of representative configurations for each adsorbate rather than seeking to identify the global minimum. Multiple starting structures were used for each adsorbate, to obtain a representative set of adsorbed structures. The adsorbates were placed with their functional groups near the surface hydroxyls. Starting structures containing dissociated adsorbates were also considered, with the dissociated proton placed within the hydrogen bond distance from both the molecule's functional group and the surface hydroxyls; however, in all dissociated structures considered here the proton became re-attached to the molecule, i.e. the final structures were all molecularly adsorbed. The adsorbed structures are presented in the electronic supplementary material, figures S1–S5, and their energies in the electronic supplementary material, tables S1–S5. The adsorption energies of all structures are summarized in figure 2. To analyse the strength of adsorption, we compared the adsorption energies and the numbers of hydrogen bonds at the surface–adsorbate interfaces (electronic supplementary material, tables S1–S5).

**Figure 3.**
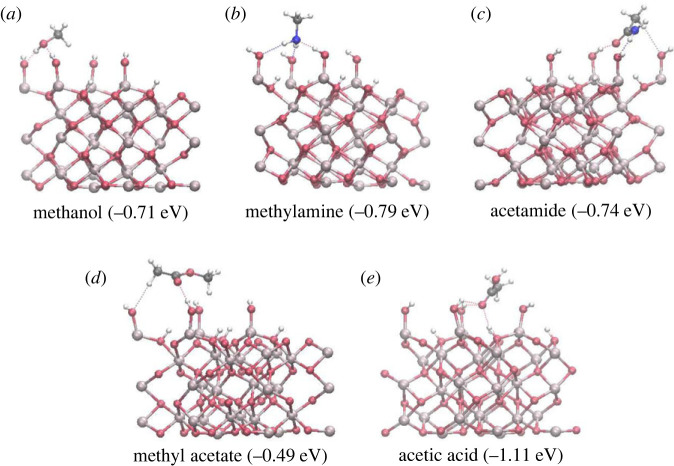
The most strongly adsorbed configurations of (*a*) methanol, (*b*) methylamine, (*c*) acetamide, (*d*) methyl acetate and (*e*) acetic acid molecules on the hydroxylated α-Al_2_O_3_ (0001) surface. The adsorption energies of these configurations are given in brackets. Red spheres—O atoms, light grey—Al, dark grey—C, blue—N, white—H atoms. Hydrogen bonds are shown with thin dashed lines. (Online version in colour.)

For methanol adsorption, five optimized structures were obtained (electronic supplementary material, figure S1), which were all molecularly adsorbed (in the initially dissociated structure methanol-1, the dissociated proton re-attached to the molecule during optimization). The energies and hydrogen bond distances of the final structures are presented in the electronic supplementary material, table S1. Methanol adsorbed on the hydroxylated α-Al_2_O_3_ (0001) surface through hydrogen bonds between the adsorbate and the hydroxyl groups of the surface. The three lowest-energy structures were very similar to each other, with the adsorption energies of −0.71 to −0.70 eV and with two 1.78–1.88 Å long surface–adsorbate hydrogen bonds between the OH group of methanol and two nearby surface hydroxyls. The most stable structure is shown in figure 3*a*. Two of these structures additionally had relatively short CH…O distances between the methyl hydrogens and oxygen atoms of the nearby surface hydroxyls (2.47–2.49 Å), which can be interpreted as weak hydrogen bonds [45]; however, the adsorption energies of the structures with and without CH…O hydrogen bonds were very similar, within 0.1 eV from each other; therefore, the energy of a CH…O bond is likely to be very small. The structure methanol-4 had only one surface–adsorbate hydrogen bond and was less strongly adsorbed, with the adsorption energy of only −0.42 eV. Thus, the potential energy surface for methanol on the hydroxylated α-Al_2_O_3_ (0001) surface has multiple deep minima of around −0.70 eV, but shallower minima (such as methanol-4) are also possible. The least strongly adsorbed structure methanol-5 (initially placed with the methyl group pointing away from the surface) had the adsorption energy of only −0.09 eV and had no surface–adsorbate hydrogen bonds, and was adsorbed only through dispersion interactions. These results allow us to conclude that hydrogen bonding is essential for strong adsorption of methanol on the hydroxylated α-Al_2_O_3_ (0001) surface, and the most likely adsorption configurations for methanol and other alcohols involve a pair of surface–adsorbate hydrogen bonds, and possibly additional weak hydrogen bonds involving hydrogens of the alkyl group. The contribution of dispersion to the overall methanol-surface interaction is likely to be of the order of −0.1 eV, similar to the adsorption energy of the structure methanol-5. Based on these adsorption energies, we can also evaluate the average strength of the surface–adsorbate O…H hydrogen bond as approximately −0.3 eV. This is larger than the −0.22 to −0.21 eV binding energies of the water dimer found in CCSD(T) and MP2 calculations [46,47]. The greater hydrogen bond strength of the adsorbed methanol compared with the isolated water dimer suggests that the surface–adsorbate interactions, where multiple hydrogen bonds are present and the adsorbate acts both as the hydrogen-bond donor and hydrogen-bond acceptor, may be stabilized by resonance-assisted hydrogen bonding observed in cyclic hydrogen-bonded dimers [48].

For methylamine, five adsorption configurations were considered, which resulted in four distinct final configurations (electronic supplementary material, figure S2 and table S2). As with methanol, all final structures were molecularly adsorbed, even if they started as dissociated structures. The most stable final structure (shown in figure 3*b*), with the adsorption energy of −0.79 eV, involved three hydrogen bonds between the adsorbate and the surface: one hydrogen bond between the amine nitrogen and a surface hydroxyl hydrogen (1.76 Å), and two hydrogen bonds between the amine hydrogens and surface hydroxyl oxygens (2.16 and 2.20 Å). The less strongly bound structures methylamine-2 and methylamine-3 with the adsorption energies of −0.59 to −0.58 eV had only two surface–adsorbate hydrogen bonds: one hydrogen bond through the amine nitrogen (1.76–1.79 Å) and one hydrogen bond through one of the amine hydrogens (1.95–2.04 Å). Structure methylamine-3 also had a possible weak hydrogen bond between methylamine's alkyl hydrogen and a surface hydroxyl oxygen; however, this bond did not additionally stabilize this structure compared with methylamine-2, showing that hydrogen bonding through alkyl groups is very weak (although alkyl groups are still likely to contribute to adsorbate binding through dispersion interactions). The weakest adsorbed configuration identified here, methylamine-5, was adsorbed to the surface through only one hydrogen bond and had the adsorption energy of −0.16 eV. Thus, similar to methanol, methylamine adsorbs on the hydroxylated α-Al_2_O_3_ (0001) surface by forming multiple surface–adsorbate hydrogen bonds. The adsorption energies for the most stable methylamine adsorbed structures, −0.79 to −0.58 eV, are similar to methanol adsorption energies of −0.71 to −0.70 eV, consistent with the similar nature of the surface–adsorbate bonding. Methylamine is able to adsorb more strongly than methanol, because it can form three hydrogen bonds to this surface, while methanol can form only two strong hydrogen bonds.

Carbonyl compounds (amides, esters and particularly carboxylic acids) were found in our previous study [32] to adsorb strongly on the bare α-Al_2_O_3_ (0001) surface, because they were able to form multiple bonds (both covalent and hydrogen bonds) to the surface; covalent bonding through the carbonyl oxygen was found to be the strongest, stronger than bonding through the other oxygen or nitrogen. Similarly, carbonyl compounds on the hydroxylated α-Al_2_O_3_ (0001) surface are likely to produce a complex adsorption potential energy landscape, with multiple possible hydrogen bonds present. We considered acetamide, methyl acetate and acetic acid as model carbonyl compounds on the hydroxylated α-Al_2_O_3_ (0001) surface.

Acetamide's most stable adsorption configuration (shown in figure 3*c*), obtained by optimization from several starting structures, involved two strong hydrogen bonds: between the carbonyl oxygen and the surface hydroxyl hydrogen (1.74 Å) and between the amide hydrogen and the surface hydroxyl oxygen (1.85 Å). A very weak hydrogen bond (2.49 Å) from the adsorbate's methyl hydrogen to a nearby surface hydroxyl could also be found. This configuration, with the adsorption energy of −0.74 eV, was slightly more strongly adsorbed that the configurations 2 and 3 (adsorption energies of −0.70 to −0.67 eV) where the amide group formed three hydrogen bonds to the surface, involving the carbonyl oxygen and the amide nitrogen and hydrogen (see the electronic supplementary material, figure S3 and table S3). Although the latter two structures formed three hydrogen bonds, those bonds were generally longer: 1.77–1.90 Å, and as long as 2.20–2.22 Å for the bonds involving the amide nitrogen, indicating that these bonds were more strained. Moreover, while configuration 1 had the methyl group close to the surface, configurations 2 and 3 had the methyl group pointing away from the surface and therefore unable to contribute to the binding through dispersion interaction. The least strongly adsorbed structure found for acetamide had the adsorption energy of −0.55 eV and had two surface–adsorbate hydrogen bonds. Again, the hydrogen bonds were rather long (1.81 and 2.03 Å), and the methyl group was pointing away from the surface. Thus, acetamide was able to bind to the hydroxylated α-Al_2_O_3_ (0001) surface with the adsorption strength similar to methylamine adsorption (up to −0.74 eV). Hydrogen bonding was the key mechanism for the amide adsorption, but the number of hydrogen bonds was not the only significant factor; the positioning of the alkyl group of the amide and its dispersion interaction with the surface also played a role.

Methyl acetate contains two oxygen atoms: the carbonyl oxygen and the ether oxygen, both of which may form hydrogen bonds to the surface hydroxyls of α-Al_2_O_3_. Two of the obtained adsorbed configurations had both of these oxygens forming hydrogen bonds to the surface (configurations 3 and 4, see the electronic supplementary material, figure S4 and table S4), with the adsorption energies of −0.43 and −0.37 eV. However, these configurations were not the most strongly adsorbed ones; the most strongly bound configurations (configuration 1 shown in figure 3*d* and configuration 2 shown in the electronic supplementary material, figure S3) contained only one surface–adsorbate hydrogen bond each, with hydrogen bonding through the carbonyl oxygen slightly more preferable than through the ether oxygen (adsorption energies of −0.49 and −0.47 eV, respectively). The low stability of the structures with two interfacial hydrogen bonds can be attributed to the effect of the relatively bulky methyl groups: to avoid steric clash with surface hydroxyls, the methyl groups of methyl acetate in configurations 3 and 4 adopted an eclipsed conformation; therefore, the energy gain of the pair of hydrogen bonds was counteracted by the energy cost of the unfavourable eclipsed conformation. In another two of the considered adsorption configurations, the bulkiness of the methyl groups prevented methyl acetate from approaching the surface close enough to form strong hydrogen bonds (structures 5 and 6, with the adsorption energies of −0.25 to −0.19 eV and with the shortest hydrogen bond of 2.14 Å). Finally, adsorption configuration 7 was structurally similar to configuration 1: both configurations were hydrogen-bonded through the carbonyl oxygen, but the more stable configuration 1 had the adsorbate in the staggered conformation, while the less stable configuration 7 (adsorption energy of −0.11 eV) had the adsorbate in the eclipsed conformation, again showing that the positions of the methyl groups of the adsorbate have a significant effect on the stability of the adsorption configurations. These results show that adsorption of esters on the hydroxylated α-Al_2_O_3_ (0001) surface takes place on a very complex potential energy landscape and that the strength of interfacial hydrogen bonds is not the only significant factor determining the strength of adsorption. Presence of alkyl groups and the conformation of the adsorbate is also likely to impact on the strength of adsorption, both through dispersion interactions and through steric constraints. In particular, this is likely to be a significant factor for adsorption of larger molecules, such as polysaccharides, on hydroxylated mineral surfaces.

Carboxylic acids similarly can bind to the hydroxylated surface through the carbonyl oxygen and through the acid's OH group. We found acetic acid to be the most strongly adsorbed small molecule in this study, and it was able to form a variety of adsorption configurations (see the electronic supplementary material, figure S5 and table S5). In its most strongly adsorbed configuration shown in figure 3*e*, with the adsorption energy of −1.11 eV, it formed two short strong hydrogen bonds: a 1.49 Å long hydrogen bond between the carboxylic hydrogen and the oxygen of the surface hydroxyl, and a 1.55 Å long hydrogen bond between the carbonyl oxygen and the surface hydrogen attached to a subsurface oxygen. These hydrogen bonds are shorter, and therefore stronger, than the hydrogen bonds formed by the adsorbed alcohol, amine, amide and ester molecules. In addition, in this configuration acetic acid formed a hydrogen bond between its methyl hydrogen and a surface hydroxyl oxygen: at 2.26 Å, this bond was longer than hydrogen bonds formed by methyl groups of the alcohol, amine, amide and ester. These short strong hydrogen bonds are responsible for the strong adsorption of acetic acid.

In addition to this most stable configuration, multiple other strongly adsorbed configurations of acetic acids were found, as shown in the electronic supplementary material, figure S5 and table S5. These structures had either three or two hydrogen bonds involving the carbonyl oxygen, the acid hydroxyl oxygen and the carboxylic hydrogen, with the adsorption energies of −0.98 to −0.93 eV. Thus, the potential energy surface for carboxylic acids adsorption is very complex, with multiple minimum-energy structures with similar stabilities. Shallower energy minima were also found (−0.55 to −0.45 eV), when the molecule was oriented in such a way that only the carbonyl group was available for hydrogen bonding to the surface.

**Figure 4.**
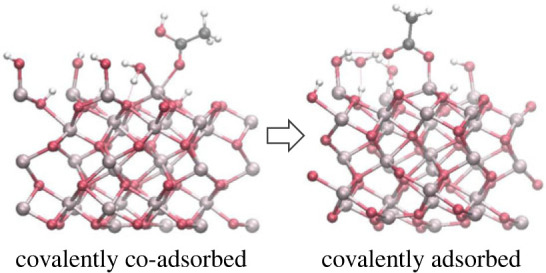
Left: acetic acid and a hydroxyl group co-adsorbed by covalent bonds to the same Al atom on the hydroxylated α-Al_2_O_3_ (0001) surface. Structure C2 is shown, where acetic acid is bound via its carbonyl oxygen. Right: acetic acid covalently bonded to the surface Al atom via its carbonyl oxygen, with the surface hydroxyl group desorbed in the form of a water molecule, which remains hydrogen-bonded to the surface. (Online version in colour.)

Interestingly, when acetic acid was placed too close to the surface in the initial structure, a different type of the adsorbed configuration was obtained where the acid molecule was covalently bonded to the surface (structures C1–C3 in the electronic supplementary material, table S6 and figure S6; example structure C2 is shown in figure 4). In these covalently bonded configurations, both the acetic acid and the surface hydroxyl were co-adsorbed to the same surface aluminium atom. The acid could adsorb either through its carbonyl oxygen or its hydroxyl oxygen, with very similar adsorption energies of −0.64 to −0.56 eV. Notably, in some of these structures the co-adsorbed surface hydroxyl picked up a hydrogen atom from a nearby hydroxyl group on the surface, transforming into a co-adsorbed water molecule (e.g. structure C2 in the left panel of figure 4). The Al–O covalent bonds were slightly shorter for the co-adsorbed hydroxyl (1.86 Å) than for the co-adsorbed water (1.93–1.98 Å) and for the co-adsorbed acid (1.99–2.08 Å). However, these distances are longer than the Al–O distance of 1.71 Å for the hydroxyl groups on the clean hydroxylated surface without adsorbates. This suggests that the co-adsorbed groups are relatively less strongly bound to their Al atom than the regular surface hydroxyls. This co-adsorption can provide a route for ligand exchange, the mechanism that is believed to operate in sorption of organic matter on soil minerals, whereby organic ligands replace hydroxyl or water ligands which are chemically bonded to surface metal ions [3]. Our results suggest that adsorbates such as carboxylic acids could co-adsorb on an Al atom which is already hydroxylated, and then either the acid would desorb again (reverting to the reactant, i.e. the acid adsorbed through hydrogen bonding), or the hydroxyl would desorb, leading to the strong covalent binding of the organic molecule to the mineral surface. The slightly longer Al–O bond for the adsorbed water (1.93–1.98 Å) than for the adsorbed hydroxyl (1.86 Å) suggests that this proton transfer to the co-adsorbed hydroxyl would facilitate the desorption of the hydroxyl and the binding of the acid molecule.

The adsorption energies of these covalently bonded co-adsorbed configurations were between −0.64 and −0.56 eV, which is weaker than the typical adsorption energies of the hydrogen-bonded acetic acid (−1.11 to −0.93 eV). Therefore, these covalently bonded co-adsorbed configurations can be viewed as intermediates in the reaction of exchange of hydroxyl ligands for acid ligands. To verify that the expected final product of this ligand exchange (the covalently adsorbed acid) is energetically favourable, we calculated the structure where acetic acid is covalently bonded to a surface Al atom, and a water molecule is hydrogen-bonded nearby (right panel in figure 4, and structure P1 in the electronic supplementary material, table S6). This structure had the adsorption energy of −1.08 eV, very close to the most stable hydrogen-bonded acid on the intact hydroxylated surface. Therefore, the hydrogen-bonded acid and the covalently adsorbed acid can be seen as the reactant and the product in the reaction of ligand exchange, separated by the less favourable intermediate with two co-adsorbed ligands. This conversion of hydrogen-bonded organic adsorbates into covalently bonded organic adsorbates is significant for the binding of organic molecules on minerals, because covalently bonded organic molecules would be less likely to desorb from the mineral surface and therefore would be more likely to be stable in soil.

Overall, our calculations showed that the typical organic functional groups, such as alcohol, amine, amide, ester and carboxylic acid, adsorb on the hydroxylated α-Al_2_O_3_ (0001) surface by hydrogen bonding, and these molecules adsorb molecularly, i.e. without dissociation. Carboxylic acid was found to be the strongest adsorbate on this surface, with adsorption energies up to −1.11 eV, but amine, amide and alcohol were also adsorbed strongly, with the best adsorption energies between −0.79 and −0.70 eV. By contrast, ester did not adsorb strongly because of the steric hindrance of the methyl groups, the factor that is likely to be more significant for adsorption of larger organic and bio molecules. This trend in the strengths of adsorption was confirmed by additional calculations of the most stable structures for each adsorbate using different density functionals and different models for describing van der Waals interactions (electronic supplementary material, figure S7 and table S7). Calculations using PW91 and B3LYP functionals with the D3 dispersion correction and using van der Waals density functional vdW-DF2 showed that, while the adsorption energies were somewhat method-dependent, the trends in adsorption stabilities were independent of the method: carboxylic acid was the most strongly adsorbed, followed by amine, amide and alcohol with very similar adsorption energies, while the ester was the least strongly adsorbed.

Interestingly, this trend in adsorption stabilities is similar to the trend observed on the bare α-Al_2_O_3_ (0001) surface, [32] and similar to the findings of Blanck *et al*. [31] who found similar orders of stabilities of organic adsorbates on the dry and hydroxylated γ-Al_2_O_3_ (100) surfaces, even though the nature of adsorption on the bare and hydroxylated surface is different: hydrogen bonding on the hydroxylated surface, and covalent bonding on the bare α-Al_2_O_3_ (0001) surface. This shows that the same key factors control the stability of adsorption on both types of surfaces: the electronegativity of adsorbates' atoms and the possibility of forming multiple surface–adsorbate bonds. Furthermore, since many mineral surfaces are hydroxylated under realistic environmental conditions [3,49], our results on adsorption on the hydroxylated α-Al_2_O_3_ (0001) surface are broadly applicable to other hydroxylated mineral surfaces: we can expect that organic and biomolecules containing acid, amine, amide and alcohol groups would adsorb strongly not only on α-Al_2_O_3_ (0001) but also on other hydroxylated mineral surfaces.

### (b) Adsorption of polysaccharide fragments on the hydroxylated α-Al_2_O_3_ (0001) surface

**Figure 5.**
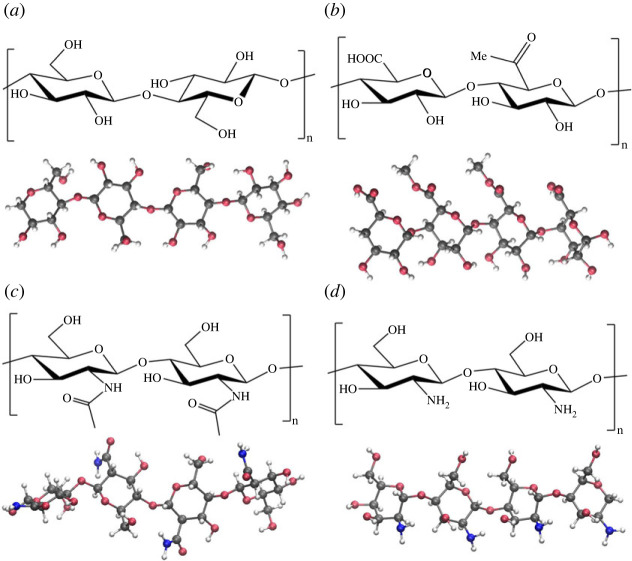
Chemical structures of polysaccharides: (*a*) cellulose, (*b*) pectin, (*c*) chitin and (*d*) chitosan, and images of their oligomers modelled in this work. Grey spheres—C atoms, red—O atoms, blue—N atoms, white—H atoms. (Online version in colour.)

To investigate the adsorption of biopolymers on the α-Al_2_O_3_ soil mineral, we modelled short oligomers of polysaccharides cellulose, chitin, chitosan and pectin on the hydroxylated α-Al_2_O_3_ (0001) surface. Polysaccharides are some of the key components of soil organic matter, alongside lignin, humin, humic and fulvic acids, and are produced by humification of plant or animal litter [5]. In particular, cellulose is found in cell walls of plants and is the most abundant organic polymer in nature [50]. It is composed of chains of d-glucose units—six-membered oxane rings with hydroxyl substituents (figure 5*a*). Pectin also is found in cell walls of plants, particularly in fruit [51]. It is structurally similar to cellulose and is composed of chains of d-galacturonic acid units—six-membered oxane rings with carboxylic acid substituents; with some of these acid groups converted to methyl esters (figure 5*b*). Chitin is the second most abundant polysaccharide on Earth, and the most abundant aminopolysaccharide; it is found in cell walls in fungi, and in exoskeletons of insects and crustaceans [52]. Like cellulose and pectin, it is composed of chains of oxane rings with hydroxyl and amide (acetylamine) group substituents (figure 5*c*). Closely related to chitin is chitosan, which is produced by deacetylation of chitin. In chitosan, the amide groups are converted to amine groups (figure 5*d*); as a result, chitosan is soluble in water and in acidic solutions [52,53].

**Figure 6.**
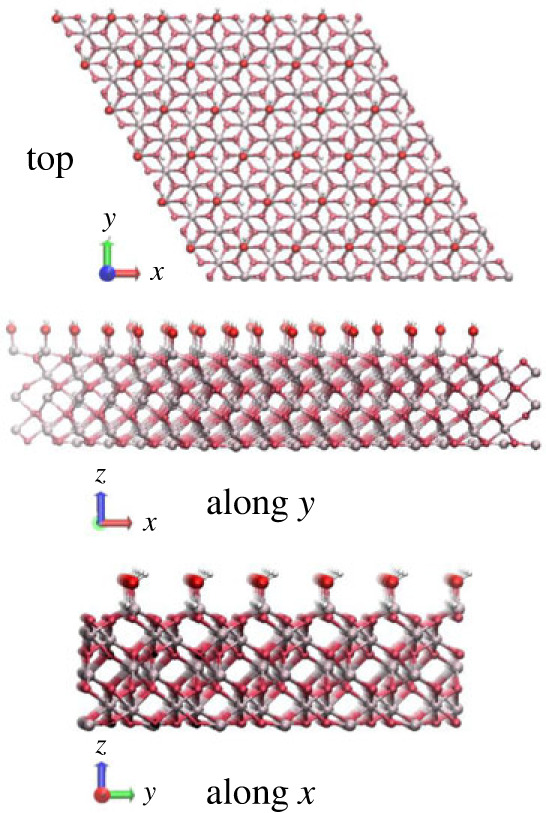
Top view and side views of the α-Al_2_O_3_ (0001)-oriented hydroxylated slabs. Slabs 6 × 6 extended in the horizontal dimensions are shown, which were used to model the adsorption of polysaccharides. (Online version in colour.)

For each of these biopolymers, a fragment containing four oxane rings was considered, as shown in figure 5. These polysaccharide molecules contain different types of functional groups: alkyl, ether and hydroxyl in cellulose; alkyl, ether, hydroxyl, ester and acid in pectin; alkyl, ether, hydroxyl and amide in chitin; alkyl, ether, hydroxyl and amine in chitosan. Thus, all of these molecules contain functional groups that were able to form strong surface–adsorbate hydrogen bonds in our study of adsorption of small organic molecules—building blocks of biomolecules. In this section, we investigated the adsorption of polysaccharide molecules on the hydroxylated α-Al_2_O_3_ (0001) surface through multiple functional groups. To obtain a representative set of structures, we considered 12 different placements of each adsorbate on the surface (except chitin where fewer positions were considered, as discussed below). Different positions were considered, either along the *x*- or along the *y*-direction on the surface. In the *y*-direction, the surface hydroxyl groups are uniformly placed (figure 6), while in the *x*-direction, surface hydroxyl groups form rows; therefore, the long-chain adsorbate can be placed either above these rows of hydroxyls or above the grooves between these hydroxyl rows. Different orientations of the adsorbates were also considered, placed either flat on the surface (i.e. the oxane rings approximately parallel to the surface) or with an edge of the molecule pointing down (i.e. the adsorbate's functional groups pointing to the surface).

**Figure 7.**
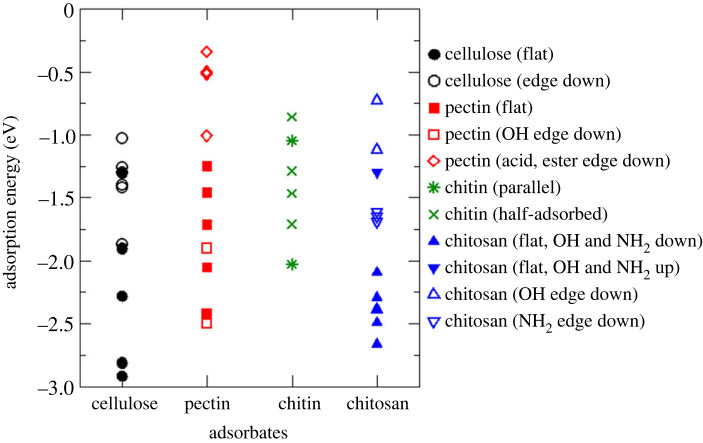
Adsorption energies of cellulose, pectin, chitin and chitosan oligomers on the hydroxylated α-Al_2_O_3_ (0001) surface. Filled symbols correspond to the molecules adsorbed with their oxane rings flat on the surface, while empty symbols correspond to the edges of the oligomers facing the surface. Further structural details of the adsorption configurations are described in the text. (Online version in colour.)

The optimized adsorption configurations of these polysaccharide oligomers are presented in the electronic supplementary material, figures S8–S11, and the adsorption energies and hydrogen bond distances are listed in the electronic supplementary material, tables S8–S11. All polysaccharides adsorbed on the hydroxylated α-Al_2_O_3_ (0001) surface by forming multiple hydrogen bonds. The energies of all adsorption configurations are summarized in figure 7. It can be seen that there is a broad range of adsorption energies obtained for each polysaccharide. To understand the relationship between the nature and strength of adsorption, the adsorption configurations of each polysaccharide are discussed below in terms of the adsorbates' orientations and the number of hydrogen bonds in the surface–adsorbate systems.

**Figure 8.**
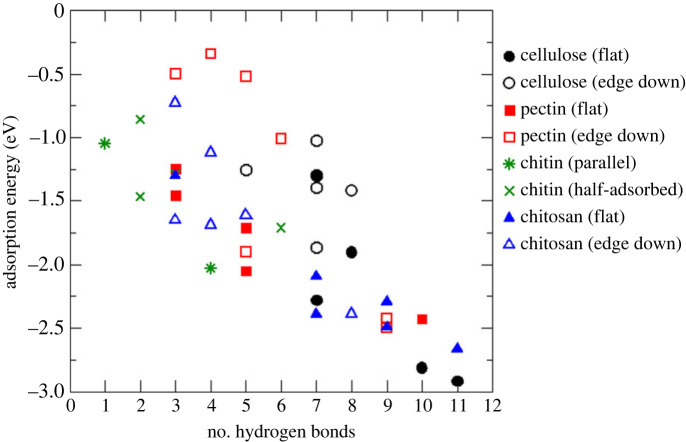
Correlation of adsorption energies of cellulose, pectin, chitin and chitosan oligomers on the hydroxylated α-Al_2_O_3_ (0001) surface with the numbers of hydrogen bonds. Filled symbols correspond to the molecules adsorbed with their oxane rings flat on the surface, while empty symbols correspond to the edges of the oligomers facing the surface. (Online version in colour.)

Cellulose had the most strongly adsorbed configuration out of all the polysaccharide oligomers studied here, with the adsorption energy of −2.91 eV, as well as several less strongly adsorbed configurations. The adsorption configurations of cellulose are shown in the electronic supplementary material, figure S8, and the adsorption energies and hydrogen bond distances are listed in the electronic supplementary material, table S8. Cellulose is composed of glucose units, i.e. oxane rings connected by ether oxygens and functionalized by hydroxyl groups, which are capable of hydrogen bonding with surface hydroxyls. Therefore, the cellulose oligomer was able to form multiple hydrogen bonds with the hydroxylated α-Al_2_O_3_ (0001) surface through its hydroxyl groups, with the adsorption energies ranging between −2.91 and −1.03 eV for the configurations modelled in this work. As seen in the electronic supplementary material, table S8, the number of hydrogen bonds formed varied greatly: between five and 11 bonds depending on the adsorption configuration, and was usually smaller than the number of functional groups that were available and were facing the surface. As seen in the summary figure 7, the adsorption was stronger when the cellulose oligomer was placed flat on the surface than when the molecule's edge (hydroxyl groups) was facing the surface. Consistent with this, the hydrogen bonding data in the electronic supplementary material, table S8, show that the molecule was able to form more hydrogen bonds when flat on the surface than when placed edge down. Adsorption energies plotted against the number of hydrogen bonds in figure 8 show that there is an obvious correlation between the adsorption energies and the number of hydrogen bonds. This, the higher stability of the flat adsorbed configurations of cellulose can be attributed to the formation of multiple hydrogen bonds, as well as to a greater contribution of dispersion interactions when multiple atoms of the molecule face the surface in the ‘flat’ configuration. Notably, the adsorption energies were smaller in the absolute value than the sum of individual hydrogen bond energies (approx. −0.3 eV per hydrogen bond for adsorbed methanol, as found in the previous section), which suggests that not all hydrogen bonds formed are equally strong and that there may be an energy cost due to deformation of the molecule when it is adsorbed on the surface.

Pectin was also adsorbed either flat or edge down, with the configurations shown in the electronic supplementary material, figure S9, and the adsorption energies and hydrogen bonding data reported in the electronic supplementary material, table S9. Pectin has several types of functional groups present: hydroxyl, acid and ester groups attached to the oxane rings. Unlike cellulose, the pectin oligomers in our model have two chemically different edges: an edge that exposes hydroxyl groups and an edge that exposes carboxylic acid and ester groups. It was expected, based on the largest stability of adsorption of carboxylic groups found in the first part of our study, that pectin would adsorb strongest with its carboxylic edge pointing towards the surface. However, this orientation was found to be the least favourable (figure 7 and the electronic supplementary material, table S9), because the slightly bulkier methyl ester groups prevented a close approach of the carboxylic groups to the surface, thus making this type of configuration the least favourable among all polysaccharides studied here, with the adsorption energies of only −1.01 to −0.34 eV. It is still possible that a different conformation of pectin, with carboxylic groups away from ester groups, would bind strongly through carboxylic groups. But in our study, the strongest types of configurations were those where the molecule lied flat on the surface and the edge down configurations where the molecule's hydroxyl groups faced the surface and formed hydrogen bonds with the surface. Their adsorption energies of −2.43 to −1.25 eV and −2.50 to −1.90 eV, respectively, are comparable with the adsorption energies of cellulose which also formed multiple hydrogen bonds via hydroxyl groups. Interestingly, multiple intramolecular bonds were formed in the adsorbed pectin oligomer, in addition to the surface–adsorbate hydrogen bonds; these hydrogen bonds likely provided additional stability to pectin adsorption configurations.

Chitin, by contrast, showed the weakest adsorption, with adsorption energies between −2.05 and −0.88 eV and smaller numbers of surface–adsorbate hydrogen bonds than seen for other adsorbates (electronic supplementary material, figure S10 and table S10). This is caused by the unusual conformation of the chitin oligomer: unlike cellulose and pectin, which formed straight chains with all oxane rings roughly in the same plane, the chitin oligomer even in the isolated state was twisted, and its oxane rings were not parallel to each other (figure 5*c*). As a consequence, it was not possible to create well-defined adsorption configurations where chitin was either adsorbed flat or with specific types of functional groups facing the surface. Moreover, because the chitin oligomer was not straight, in several adsorption configurations only one end of the chitin molecule was in contact with the surface (see the electronic supplementary material, figure S10). Therefore, the studied chitin adsorption configurations were classified into parallel (where functional groups of all rings were in contact with the surface) and half-adsorbed (where only two rings were in contact with the surface). Parallel adsorption provided the strongest found adsorption energy for chitin (−2.03 eV); however, there was no systematic trend of parallel configurations being more strongly bound than half-adsorbed configurations (figures 7 and 8 and the electronic supplementary material, table S10). Instead, there was a clear correlation between the adsorption energies and the number of hydrogen bonds in the surface–adsorbate system. Overall, these results show that chitin adsorption on the hydroxylated α-Al_2_O_3_ (0001) surface is not as favourable as other polysaccharides, because its conformation does not facilitate multiple hydrogen bonds to the surface.

Finally, the adsorption of chitosan, a derivative of chitin, was modelled, with the structures presented in the electronic supplementary material, figure S11, and energies and hydrogen bonding information in the electronic supplementary material, table S11. As with all polysaccharides, there was a very clear correlation between the adsorption energies and the numbers of hydrogen bonds, as seen in figure 8. The conformation of the chitosan oligomer used in this study is such that one edge of the molecule is hydroxyl-functionalized and the other edge is amine-functionalized. When adsorbed flat on the surface, the amine and alcohol groups can either point towards the surface or point upwards. The flat adsorbed configurations where the functional groups pointed towards the surface were systematically the most stable ones and were among the most stable adsorption configurations of all polysaccharides (figure 7), while the flat adsorption with functional groups pointing up and the amine edge down adsorption configurations were less favourable, although usually still stronger than the adsorption of chitin. Hydroxyl edge down configurations were typically the least favourable for chitosan. Once again, these results show that the numbers of hydrogen bonds in the surface–adsorbate systems are a key factor in the stability of adsorption, but it is also clear that the nature of hydrogen bonds also matters: for example, amine groups helped achieve strong adsorption.

To summarize, the polysaccharide molecules were found to adsorb strongly on the hydroxylated α-Al_2_O_3_ (0001) surface by forming multiple hydrogen bonds with the surface. Numerous stable adsorption configurations were found for each adsorbate, which show that potential energy surfaces of biopolymer adsorption are very complex, with many configurations likely to coexist and interconvert. Therefore, simulations of large numbers of adsorption configurations are needed to obtain a comprehensive view of the nature and strength of adsorption of biopolymer. Our calculations show that the number of hydrogen bonds formed at the interface is the key factor that determines the stability of adsorption.

## 4. Conclusion

In this work we modelled the adsorption of small organic molecules—building blocks of naturally occurring biomolecules—on the hydroxylated α-Al_2_O_3_ (0001) surface as a model soil mineral surface, to investigate the trends that govern the binding or organic carbon to soil minerals under realistic environmental conditions. We found that alcohol, amine, amide, ester and carboxylic acid groups could adsorb on this surface through hydrogen bonding, with multiple adsorption configurations possible, preferring to maximize the number of surface–adsorbate hydrogen bonds. Carboxylic acid was the strongest adsorbate, similar to what was found earlier for small organic molecules on the bare (non-hydroxylated) Al-terminated α-Al_2_O_3_ (0001) surface [32], despite the different nature of its bonding on the two surfaces: hydrogen bonding on the hydroxylated surface and covalent bonding with some additional hydrogen bonding on the bare surface. Amine, amide and alcohol also formed strong hydrogen bonds with the hydroxylated α-Al_2_O_3_ (0001) surface. Thus, organic and biomolecules containing carboxylic acid, amine, amide and alcohol functional groups are likely to bind strongly to the surface of the α-Al_2_O_3_ mineral in soil under realistic environmental conditions when the surface of this mineral is hydroxylated.

Our calculations also showed that the presence of bulky functional groups, such as alkyls in methyl ester, may hinder binding by preventing other, stronger-binding functional groups from closely approaching the surface. We also showed how the strongly binding carboxylic groups can adsorb on the surface by covalent bonding, by forming adsorption configurations where both the carboxylic group and a surface hydroxyl are covalently bonded to the same surface Al atom. Such structures can be seen as intermediates in the process of ligand exchange [3], where hydroxyls on the mineral surface are exchanged for organic ligands, resulting in strong covalent bonding of organic molecules to minerals.

To obtain a more comprehensive understanding of the binding or organic carbon to soil minerals, we modelled the adsorption of complex naturally occurring biopolymers on the hydroxylated α-Al_2_O_3_ (0001) surface. We considered natural biopolymers—polysaccharides—which originate from plant litter (cellulose and pectin) or animal litter (chitin), or biopolymers which can be produced by chemical modification of natural biopolymers (chitosan produced from chitin). These biopolymers are likely to be naturally present in soils or are easily available as waste products of the food processing industry and can be added to soil. All of these biomolecules were able to bind to the surface in a variety of adsorption configurations, by forming multiple hydrogen bonds. However, our calculations showed that adsorption of these biomolecules is not a simple sum of their building blocks—functional groups. The number and the nature of available functional groups is not the only factor that controls their adsorption. The other key factor is the number and strength of hydrogen bonds that can be formed at the surface–adsorbate interface. Flexibility of molecular conformations, which enables these molecules to achieve multiple hydrogen bonds with the surface, is also important, as evidenced by the strong adsorption of cellulose, pectin and chitosan, and less favourable adsorption of chitin whose twisted conformation did not allow formation of multiple surface–adsorbate bonds. This flexibility of molecular conformations, combined with the thermal motion at ambient temperature, is likely to result in structural fluctuations, which would enable the adsorbates to convert between different adsorption configurations. Accessibility of multiple adsorption configurations is likely to increase the entropy of adsorption, thus driving the entropic contribution to the free energy of adsorption and additionally stabilizing the adsorption.

There is scope for more in-depth investigations of organic and biomolecules adsorption on minerals, e.g. to consider the effect of pH and protonation of functional groups such as amine, and deprotonation of carboxylic acid groups, which may affect their strength of binding. Further investigations of the mechanism and activation energies for the conversion from hydrogen-bonded to chemisorbed adsorbates would provide information on the rates of ligand exchange, the process that leads to strong covalent bonding of organic adsorbates. Moreover, since water is normally present in the soil environment, the modelling of adsorption could be extended from the hydrated mineral/vacuum interface modelled here to the hydrated mineral/water interface, using either an explicit or implicit water solvent model. The data on the multiple adsorption configurations obtained in this study can be used to fit an alumina/organic potential, which may be used in combination with the ClayFF potential [54] for the mineral component and a water potential to model alumina/organic adsorbate/water interfaces. We expect that the adsorption of biomolecules at the mineral/water interface would be less strong than at the dry interface because free (unadsorbed) molecules would be stabilized by the solvent environment, but we expect that the qualitative trends on the nature and strength of adsorption would remain similar.

Moreover, we expect that the obtained results on the nature and strengths of binding of these biomolecules and individual functional groups are transferable to other Al hydroxides and oxyhydroxides [3] and to other hydroxylated mineral surfaces, such as quartz, feldspar and mica [49], where surface hydroxyls would be available for hydrogen bonding with molecular adsorbates.

Overall, our atomic-scale results illustrate how soil organic matter that originates from plant and animal litter, such as cellulose, pectin and chitin, is preserved in soil through strong binding to soil minerals. To increase the amount of organic matter in soil, soil amendments such as organic or synthetic mulches can be suitably chosen, to contain the functional groups that strongly bind to soil minerals, such as carboxylic acid, alcohol and amine groups. This encompasses natural materials (cellulose-containing leaves, grass clippings, etc.) and chemically processed food industry waste products such as chitosan, which have the potential be added to soils to increase the amount of stably preserved organic carbon and to replenish the loss of soil organic carbon, and thus to maintain the fertility of soils.

## Data Availability

Coordinates (*xyz* format) of adsorption configurations of methanol, methylamine, acetamide, methyl acetate, acetic acid, cellulose, pectin, chitin and chitosan on the hydroxylated α-Al_2_O_3_ (0001) surface are openly available in the University of Sheffield ORDA (Online Research Data) repository at https://doi.org/10.15131/shef.data.21397485 [55]. Figures showing images of all structures and tables of energies of all structures are provided in the electronic supplementary material [56].
